# Integrating 7-day D-dimer exposure into deep vein thrombosis risk prediction after gastrointestinal surgery

**DOI:** 10.1038/s41598-025-14960-7

**Published:** 2025-08-13

**Authors:** Shuwei Weng, Chen Ding, Die Hu, Likui Huang, Daoquan Peng

**Affiliations:** 1 Department of Cardiovascular Medicine, The Second Xiangya Hospital, Central South University, Changsha, Hunan China; 2 Research Institute of Blood Lipid and Atherosclerosis, Changsha, Hunan China; 3 Department of Cardiology, The First Affiliated Hospital of Fujian Medical University, Fuzhou, Fujian China; 4 Department of Surgery, The First Hospital of Putian City, Affiliated to Putian University, Putian, Fujian China

**Keywords:** Diagnostic markers, Predictive markers, Gastrointestinal system

## Abstract

Deep vein thrombosis (DVT) is a serious complication following gastrointestinal surgery. While D-dimer is a widely used biomarker for thrombosis, its postoperative specificity is limited due to inflammatory interference. This study introduces a novel cumulative metric—7-day D-dimer exposure (7dDDE)—to quantify perioperative coagulation burden. We retrospectively analyzed 525 patients undergoing gastrointestinal surgery, performed propensity score matching, and constructed a multivariable logistic regression model incorporating 7dDDE and other clinical variables. Model performance was evaluated using ROC curves, decision curve analysis, calibration plots, SHAP values, and a nomogram. Additionally, a linear mixed-effects model assessed D-dimer trajectories over time. The results demonstrated that 7dDDE was independently associated with postoperative DVT and was the most influential predictor in the model. The model showed good discrimination and clinical utility. Longitudinal analysis further revealed significant differences in D-dimer dynamics between DVT and non-DVT groups, even after adjustment for confounders. These findings support the use of 7dDDE as a robust biomarker for thrombotic risk stratification and highlight the importance of integrating temporal biomarker patterns into perioperative DVT prediction.

**Supplementary Information:**

The online version contains supplementary material available at 10.1038/s41598-025-14960-7.

## Introduction

Venous thromboembolism (VTE), comprising deep vein thrombosis (DVT) and pulmonary embolism (PE), is the third leading cause of vascular-related mortality worldwide, following myocardial infarction and stroke^1^. It also remains one of the most common and potentially fatal postoperative complications^2,3^. Both hospitalization and surgical procedures are established risk factors for VTE, especially major operations, which increase thrombosis risk due to prolonged immobilization, endothelial injury, and surgery-induced hypercoagulable states^4,5^. Among VTE events, DVT is the predominant clinical manifestation. Although often asymptomatic, DVT can lead to PE in approximately one-third of patients, posing a significant threat to survival^6^. Therefore, early identification of high-risk individuals during the perioperative period is critical.

Current clinical guidelines recommend pharmacologic and/or mechanical thromboprophylaxis for patients at high risk^7,8^. However, indiscriminate use of anticoagulants without proper risk stratification may increase the incidence of bleeding complications, particularly in patients with low baseline thrombotic risk^9^. Thus, achieving personalized perioperative risk stratification is essential for optimizing preventive strategies.

Traditional risk assessment tools such as the Caprini score are widely used in surgical populations^10^, but they mainly rely on static preoperative variables and fail to capture dynamic physiological changes after surgery. Moreover, some studies have questioned the empirical basis of these scores, noting inconsistencies between the assigned weights and actual risk estimates^11^. These models also lack integration of time-varying biomarkers, such as coagulation and inflammatory indicators, which may provide important insights into thrombosis risk.

D-dimer, a fibrin degradation product, is a well-established biomarker of coagulation and fibrinolytic activity, and is commonly elevated in patients with acute or active DVT^12^. However, in the early postoperative phase, D-dimer levels can be confounded by surgical inflammation, reducing their specificity. Therefore, in clinical practice, D-dimer is primarily used as a rule-out rather than a confirmatory test for DVT^13^.

Emerging evidence suggests that cumulative exposure to biomarkers over time, rather than single time-point measurements, may offer superior predictive power in risk models. For example, cumulative exposure to low-density lipoprotein cholesterol (LDL-C) has been shown to be significantly associated with peripheral artery disease and offers better predictive value than LDL-C levels at any single time point^14^. Similarly, cumulative LDL-C exposure from age 18 to 60 has been independently linked to subsequent coronary heart disease, beyond midlife LDL-C levels^15^. While these findings support the utility of time-integrated biomarkers in chronic disease contexts, whether this concept can be meaningfully extended to dynamic, acutely fluctuating perioperative markers such as D-dimer remains uncertain. Therefore, caution is warranted when extrapolating from chronic risk paradigms to acute care settings, and further studies are needed to clarify the clinical relevance of cumulative D-dimer exposure in this context.

Inspired by this concept, we introduced a cumulative indicator termed 7-day D-dimer exposure (7dDDE) to quantify coagulation burden after gastrointestinal surgery. This index was calculated using the trapezoidal rule based on D-dimer levels collected preoperatively and on postoperative days 1, 3, 5, and 7^16^, thereby capturing both the intensity and duration of the hypercoagulable state. We hypothesized that 7dDDE would serve as an independent predictor of postoperative DVT. To test this, we conducted a retrospective cohort study in a Chinese surgical population, aiming to establish a predictive model incorporating 7dDDE for improved DVT risk stratification and individualized thromboprophylaxis.

## Materials and methods

### Data collection

Between January 2023 and January 2024, we retrospectively collected data from patients who underwent gastrointestinal surgery at The First Hospital of Putian, Fujian Province, China. The study protocol was approved by the Ethics Committee of The First Hospital of Putian (Approval No: 2023-121). All procedures were conducted in accordance with the Declaration of Helsinki and relevant guidelines for research involving human participants. Due to the retrospective nature of the study, informed consent was waived by the Ethics Committee of The First Hospital of Putian.

**Fig. 1:**
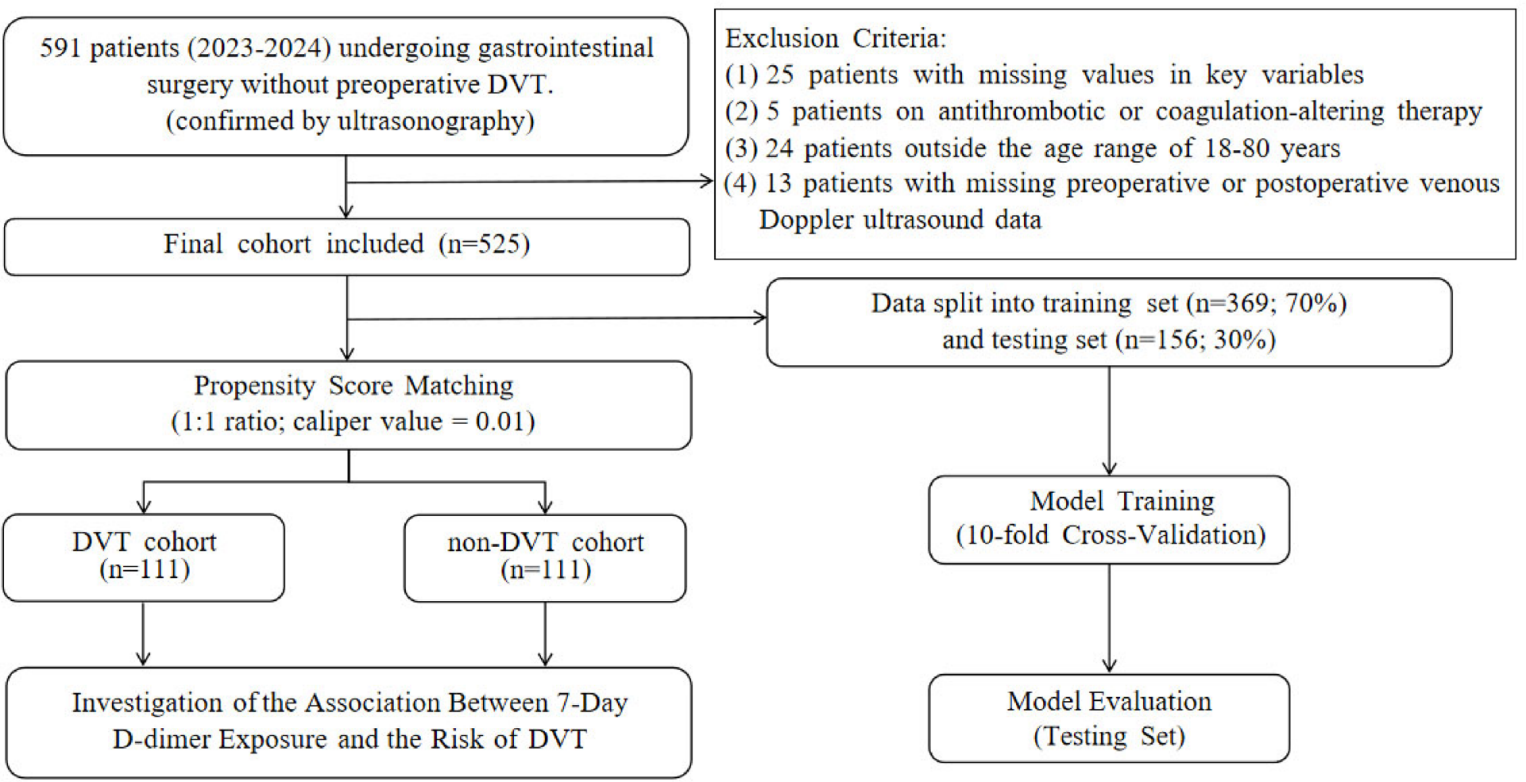
Study flowchart.

Patients were included if they met all of the following criteria: (1) aged between 18 and 80 years; (2) no evidence of preoperative DVT based on duplex ultrasonography, with available postoperative ultrasound data within 7 days after surgery; (3) complete D-dimer measurements at baseline and on postoperative days 1, 3, 5, and 7, as well as no missing values in other key clinical variables; (4) no recent use of medications known to affect coagulation or anticoagulation function. After applying the inclusion and exclusion criteria, a total of 525 patients were included in the final study cohort (Fig. 1).

### DVT in patients under gastrointestinal surgery assessment

The assessment of preoperative and postoperative DVT was performed using lower extremity venous ultrasonography. All examinations were independently conducted by two qualified attending sonographers from the Department of Ultrasound at The First Hospital of Putian. In cases of disagreement between the two assessors, the final diagnosis was determined by a senior ultrasound specialist with an associate chief physician title or above.

All patients underwent standardized bilateral lower limb venous ultrasound prior to surgery to exclude pre-existing DVT. Within 7 days postoperatively, repeat ultrasound was performed to identify newly developed DVT. The diagnostic criteria for DVT were based on the international consensus on ultrasound evaluation^17^, including the following three key imaging findings: (1) Incomplete venous compressibility under probe pressure; (2) The presence of abnormal hyperechoic intraluminal structures suggestive of thrombus; and (3) Absence or marked reduction of blood flow signals on color Doppler imaging at the suspected thrombus site. The occurrence of DVT within 7 days after surgery served as the primary outcome in this study and was used for model construction and risk prediction analysis.

### Clinical features

A total of 44 potential clinical variables available at admission were included in this study as candidate predictors for subsequent modeling, excluding DVT status. Based on D-dimer levels measured preoperatively and on postoperative days 1, 3, 5, and 7, we constructed a composite variable to reflect the dynamic thrombotic burden during the early postoperative period to 7-day D-dimer exposure. 7dDDE was defined as the area under the D-dimer concentration–time curve from preoperative to postoperative day 7. This area was approximated using the trapezoidal rule, allowing integration of serial measurements across multiple time points to represent the total cumulative D-dimer exposure during the early postoperative phase, as illustrated by the following formula:

$$7{\text{dDDE}} = \mathop \sum \limits_{{{\text{i}} = 1}}^{{\text{n}}} \frac{{\left( {{\text{D}}_{{\text{i}}} + {\text{D}}_{{{\text{i}} - 1}} } \right){*}({\text{T}}_{{\text{i}}} - {\text{T}}_{{{\text{i}} - 1}} )}}{2}$$

Here, D₀ refers to the preoperative D-dimer level, and Dₙ represents the D-dimer level on postoperative day 7. The corresponding time points are denoted as T₀ (preoperative) and Tₙ (postoperative day 7), respectively. The remaining D_i_ and T_i_ represent D-dimer levels and time points on postoperative days 1, 3, and 5.

During feature engineering, we first excluded variables with strong multicollinearity based on pairwise correlation coefficients greater than 70% and variance inflation factors exceeding 5. Subsequently, numerical variables were subjected to feature selection using both the Boruta algorithm and Lasso regression, and the intersection of the two methods was used to define the final feature set. Details of the feature selection and engineering process are shown in Supplementary Fig. 1 and Supplementary Table 1. A total of 15 variables were ultimately included in the modeling analysis, comprising 5 numerical variables and 10 categorical variables.

Given the imbalance in sample size between the DVT and non-DVT groups and the overall limited cohort size, we transformed age and BMI into ordered categorical variables—age groups and BMI groups—to improve the feasibility and quality of subsequent propensity score matching and modeling.

### Statistical analysis

All statistical analyses were performed using R software (version 4.3.0). The normality of continuous variables was assessed using the Shapiro–Wilk test, and none of the variables followed a normal distribution. Therefore, continuous variables were expressed as median (interquartile range) and compared between groups using the Mann–Whitney U test. Categorical variables were presented as counts and percentages, and compared using the chi-square test or Fisher’s exact test, as appropriate.

To control for potential confounding in the assessment of DVT occurrence, a propensity score matching (PSM) model was constructed based on sex, age group, and BMI group. Nearest-neighbor matching was applied with a 1:1 ratio and a caliper of 0.01. Covariate balance before and after matching was evaluated using standardized mean differences (SMDs), and visualized using a Love plot.

The matched dataset was used for both univariate and multivariate logistic regression analyses to examine the independent association between 7dDDE and postoperative DVT, with adjustment for relevant preoperative covariates. Subgroup analyses and interaction tests were conducted to evaluate the consistency and potential effect modification of 7dDDE across different populations.

For model development, the original dataset was randomly split into a training set and a testing set in a 7:3 ratio. A multivariable logistic regression model was built on the training set, with hyperparameter tuning performed via tenfold cross-validation. The model was validated on the testing set, where the receiver operating characteristic (ROC) curve was plotted, and the area under the curve (AUC), sensitivity, specificity, and optimal cutoff point (based on the Youden index) were calculated.

Model performance was further evaluated using decision curve analysis (DCA), clinical impact curves (CIC), and calibration plots. Model interpretability was assessed using SHAP values to rank feature importance. Finally, a nomogram was constructed based on the top five predictors to facilitate individualized risk prediction. All statistical tests were two-tailed, and a *P* value < 0.05 was considered statistically significant.

Additionally, to evaluate the dynamic trends of D-dimer levels over time and their interaction with DVT status, we performed a linear mixed-effects model (LMM) using the lmerTest package. The model included repeated D-dimer measurements at five timepoints (preoperative, postoperative days 1, 3, 5, and 7) as the dependent variable, with fixed effects of timepoint, DVT group, and their interaction, while adjusting for age, BMI, gender, and log-transformed CRP. Patient ID was specified as a random effect to account for intra-individual variability. Type III ANOVA was applied to assess the significance of fixed effects, and group-specific D-dimer trajectories were visualized using ggplot2.

## Results

### Baseline characteristics

A total of 525 patients were ultimately included in this study, comprising 112 patients with DVT and 413 without DVT. Baseline analysis revealed significant differences between the two groups in terms of age, hemoglobin level, preoperative triglycerides, postoperative C-reactive protein (CRP), postoperative estimated glomerular filtration rate, as well as D-dimer levels measured preoperatively and on postoperative days 1, 3, 5, and 7 (Table 1). Density plots of all continuous variables are presented in Supplementary Fig. 2.

**Table 1:** Comparison of clinical characteristics between the DVT and non-DVT groups.

Characteristics	Subgroup	non-DVT (n = 413)	DVT (n = 112)	*P* value
7dDDE (µg/mL·day)		17.44 [11.13, 26.89]	28.40 [19.73, 41.06]	< 0.001
Age (years)		66.00 [58.00, 70.00]	69.00 [62.00, 73.00]	0.003
BMI (kg/m^2^)		22.50 [20.30, 25.10]	22.70 [19.88, 24.20]	0.126
Gender				1
	Female	158 (38.3)	43 (38.4)	
	Male	255 (61.7)	69 (61.6)	
ASA Score				0.52
	I	11 (2.7)	2 (1.8)	
	II	270 (65.4)	72 (64.3)	
	III	130 (31.5)	36 (32.1)	
	IV_i_	2 (0.5)	2 (1.8)	
Anesthesia method				0.871
	GA with Double-Lumen Tube + Epidural	8 (1.9)	2 (1.8)	
	GA with Intubation	90 (21.8)	21 (18.8)	
	GA with Intubation + Epidural	289 (70.0)	80 (71.4)	
	GA with Intubation + Nerve Block	20 (4.8)	6 (5.4)	
	others	6 (1.5)	3 (2.7)	
Smoking history				0.225
	No	397 (96.1)	104 (92.9)	
	Yes	16 (3.9)	8 (7.1)	
Past surgical history				0.55
	No	291 (70.5)	75 (67.0)	
	Yes	122 (29.5)	37 (33.0)	
History of cardiovascular disease				1
	No	389 (94.2)	105 (93.8)	
	Yes	24 (5.8)	7 (6.2)	
History of malignancy				0.959
	No	388 (93.9)	106 (94.6)	
	Yes	25 (6.1)	6 (5.4)	
History of diabetes				1
	No	341 (82.6)	92 (82.1)	
	Yes	72 (17.4)	20 (17.9)	
History of hypertension				0.752
	No	289 (70.0)	76 (67.9)	
	Yes	124 (30.0)	36 (32.1)	
History of fracture				0.857
	No	406 (98.3)	111 (99.1)	
	Yes	7 (1.7)	1 (0.9)	
Temperature (℃)		36.50 [36.40, 36.50]	36.50 [36.40, 36.50]	0.923
Pulse (beats per minute)		73.00 [66.00, 83.00]	75.00 [68.00, 81.00]	0.992
Respiration (breaths per minute)		18.00 [18.00, 18.00]	18.00 [18.00, 18.00]	0.392
Surgery duration (hours)		6.00 [5.00, 7.00]	6.50 [5.50, 7.50]	0.233
Intraoperative blood loss (mL)		100.00 [50.00, 100.00]	100.00 [57.50, 150.00]	0.185
Intraoperative Blood transfusion (mL)		0.00 [0.00, 0.00]	0.00 [0.00, 0.00]	0.057
Fibrinogen (g/L)		3.19 [2.70, 3.78]	3.21 [2.82, 3.85]	0.087
APTT (s)		24.80 [22.70, 27.20]	24.60 [22.08, 27.21]	0.475
PT (s)		11.10 [10.70, 11.60]	11.20 [10.80, 11.70]	0.242
Hemoglobin (g/L)		124.00 [110.00, 134.00]	118.00 [100.75, 131.00]	0.029
Platelet count (× 10⁹/L)		218.00 [180.00, 266.00]	226.50 [177.75, 274.25]	0.938
Preoperative triglycerides (mmol/L)		1.17 [0.87, 1.69]	1.06 [0.80, 1.44]	0.019
Postoperative triglycerides (mmol/L)		0.73 [0.54, 1.15]	0.65 [0.50, 1.17]	0.852
Preoperative total cholesterol (mmol/L)		4.80 [4.15, 5.55]	4.76 [4.16, 5.28]	0.225
Postoperative total cholesterol (mmol/L)		3.72 [3.14, 4.42]	3.69 [3.20, 4.20]	0.27
Preoperative HDL (mmol/L)		1.40 [1.19, 1.63]	1.40 [1.17, 1.64]	0.397
Postoperative HDL (mmol/L)		1.12 [0.96, 1.30]	1.10 [0.94, 1.29]	0.22
Preoperative LDL (mmol/L)		2.80 [2.35, 3.43]	2.76 [2.36, 3.18]	0.394
Postoperative LDL (mmol/L)		2.18 [1.75, 2.61]	2.16 [1.78, 2.52]	0.526
Preoperative creatinine (μmol/L)		69.00 [56.00, 82.00]	66.00 [56.00, 80.00]	0.263
Postoperative creatinine (μmol/L)		69.00 [56.00, 81.00]	70.00 [57.00, 83.00]	0.469
Postoperative CRP (mg/L)		24.60 [15.84, 39.70]	35.70 [20.20, 63.68]	< 0.001
Preoperative D-Dimer (μg/mL)		0.38 [0.22, 0.77]	0.58 [0.32, 1.15]	0.001
Postoperative D-Dimer day1 (μg/mL)		3.07 [1.80, 4.95]	4.96 [3.06, 8.78]	< 0.001
Postoperative D-Dimer day3 (μg/mL)		2.22 [1.44, 3.84]	3.45 [2.37, 5.66]	< 0.001
Postoperative D-Dimer day5 (μg/mL)		2.33 [1.51, 3.69]	3.82 [2.57, 5.98]	< 0.001
Postoperative D-Dimer day7 (μg/mL)		2.03 [1.09, 3.46]	3.98 [2.66, 6.09]	< 0.001
Systolic Blood Pressure (mmHg)		135.00 [124.00, 150.00]	136.00 [125.00, 150.75]	0.459
Diastolic blood pressure (mmHg)		76.00 [69.00, 84.00]	75.00 [68.00, 81.00]	0.37
Preoperative eGFR (mL/min/1.73 m^2^)		91.80 [80.27, 100.23]	91.02 [81.80, 97.71]	0.52
Postoperative eGFR (mL/min/1.73 m^2^)		92.34 [80.27, 100.35]	89.03 [73.55, 97.85]	0.019

Continuous variables are presented as median [interquartile range] due to non-normal distributions, and were compared using the Mann–Whitney U test. Categorical variables are presented as counts (percentages) and compared using the chi-square test or Fisher’s exact test, as appropriate.

Abbreviations: 7dDDE, 7-day D-dimer Exposure; APTT, Activated Partial Thromboplastin Time; PT, Prothrombin Time; eGFR, Estimated Glomerular Filtration Rate.

### Analysis based on propensity score matching

To minimize confounding effects, PSM was performed between the DVT and non-DVT groups, resulting in 111 matched patients in each group. The matching was effective, as several covariates exhibited SMD greater than 0.1 before matching, whereas after matching, all covariates except for history of malignancy and history of fracture had SMDs below 0.1 (Table 2). The improved balance between groups was also visually confirmed using a Love plot (Supplementary Fig. 3).

**Table 2:** Comparison of baseline characteristics between DVT and non-DVT groups before and after propensity score matching.

Characteristics	Levels	Before PSM			After PSM		
		nonDVT (n = 413)	DVT (n = 112)	SMD	nonDVT (n = 111)	DVT (n = 111)	SMD
7dDDE (µg/mL·day)		17.44 [11.13, 26.89]	28.40 [19.73, 41.06]	0.743	18.34 [11.78, 25.18]	27.92 [19.57, 41.47]	0.657
Age group				0.336			< 0.001
	Age < 50	39 (9.4)	7 (6.2)		7 (6.3)	7 (6.3)	
	50 ≤ Age < 60	78 (18.9)	13 (11.6)		12 (10.8)	12 (10.8)	
	60 ≤ Age < 70	169 (40.9)	41 (36.6)		41 (36.9)	41 (36.9)	
	Age ≥ 70	127 (30.8)	51 (45.5)		51 (45.9)	51 (45.9)	
BMI group				0.187			< 0.001
	BMI < 18.5	43 (10.4)	14 (12.5)		13 (11.7)	13 (11.7)	
	18.5 ≤ BMI < 24	224 (54.2)	68 (60.7)		68 (61.3)	68 (61.3)	
	BMI ≥ 24	146 (35.4)	30 (26.8)		30 (27.0)	30 (27.0)	
Gender				0.003			< 0.001
	Female	158 (38.3)	43 (38.4)		42 (37.8)	42 (37.8)	
	Male	255 (61.7)	69 (61.6)		69 (62.2)	69 (62.2)	
Smoking history				0.144			0.074
	No	397 (96.1)	104 (92.9)		105 (94.6)	103 (92.8)	
	Yes	16 (3.9)	8 (7.1)		6 (5.4)	8 (7.2)	
Past surgical history				0.075			0.094
	No	291 (70.5)	75 (67.0)		69 (62.2)	74 (66.7)	
	Yes	122 (29.5)	37 (33.0)		42 (37.8)	37 (33.3)	
History of cardiovascular disease				0.018			< 0.001
	No	389 (94.2)	105 (93.8)		104 (93.7)	104 (93.7)	
	Yes	24 (5.8)	7 (6.2)		7 (6.3)	7 (6.3)	
History of malignancy				0.03			0.14
	No	388 (93.9)	106 (94.6)		101 (91.0)	105 (94.6)	
	Yes	25 (6.1)	6 (5.4)		10 (9.0)	6 (5.4)	
History of Diabetes				0.011			0.023
	No	341 (82.6)	92 (82.1)		90 (81.1)	91 (82.0)	
	Yes	72 (17.4)	20 (17.9)		21 (18.9)	20 (18.0)	
History of hypertension				0.046			0.019
	No	289 (70.0)	76 (67.9)		75 (67.6)	76 (68.5)	
	Yes	124 (30.0)	36 (32.1)		36 (32.4)	35 (31.5)	
History of fracture				0.071			0.183
	No	406 (98.3)	111 (99.1)		107 (96.4)	110 (99.1)	
	Yes	7 (1.7)	1 (0.9)		4 (3.6)	1 (0.9)	
Preoperative triglycerides (mmol/L)	1.17 [0.87, 1.69]	1.06 [0.80, 1.44]	0.291		1.22 [0.90, 1.83]	1.06 [0.80, 1.42]	0.393
Postoperative CRP (mg/L)		24.60 [15.84, 39.70]	35.70 [20.20, 63.68]	0.387	26.00 [18.00, 45.72]	36.10 [20.20, 63.75]	0.354
Surgery duration (hours)		6.00 [5.00, 7.00]	6.50 [5.50, 7.50]	0.143	6.00 [5.15, 6.95]	6.50 [5.55, 7.50]	0.352
Preoperative D-Dimer (μg/mL)		0.38 [0.22, 0.77]	0.58 [0.32, 1.15]	0.236	0.48 [0.26, 0.89]	0.58 [0.32, 1.15]	0.199

Continuous variables are presented as median [interquartile range], and categorical variables as frequency (percentage). Standardized mean differences (SMD) were used to assess covariate balance between groups, with SMD < 0.1 generally indicating acceptable balance. PSM was performed at a 1:1 ratio with a caliper value of 0.01.

Univariate and multivariate logistic regression analyses were conducted on the matched dataset (Table 3). The results showed that 7dDDE remained significantly associated with postoperative DVT after adjusting for covariates (*P* < 0.05), indicating that it is an independent predictor of DVT. Subgroup analyses (Fig.  and ﻿Supplementary Table 2 ) revealed that the association between 7dDDE and DVT risk was consistently positive across most subgroups, suggesting a robust effect. The association was not statistically significant only in patients with a history of cardiovascular disease. Further interaction analysis showed no significant interaction between 7dDDE and cardiovascular disease history (OR = 0.97, 95% CI 0.87–1.08, P = 0.563).

**Table 3:** Logistic regression analysis of feature-engineered continuous variables associated with postoperative DVT risk.

Characteristics	Univariate logistic regression		Multivariate logistic regression	
	OR(95% CI)	*P* value	OR(95% CI)	*P* value
7dDDE (µg/mL·day)	1.043 (1.025–1.065)	1.6E−05	1.043 (1.021–1.068)	2.6E−04
Preoperative triglycerides (mmol/L)	0.590 (0.396–0.827)	5.1E−03	0.574 (0.358–0.855)	1.3E−02
Postoperative CRP (mg/L)	1.012 (1.003–1.022)	1.2E−02	1.009 (0.999–1.02)	8.1E−02
Surgery duration (hours)	1.327 (1.127–1.583)	1.0E−03	1.234 (1.031–1.52)	4.0E−02
Preoperative D-Dimer (μg/mL)	1.168 (0.998–1.474)	1.5E−01	0.982 (0.892–1.212)	7.8E−01

This table includes all continuous variables selected through feature engineering (Boruta and LASSO regression). Univariate and multivariate logistic regression models were applied to assess their associations with DVT after gastrointestinal surgery.

**Fig. 2:**
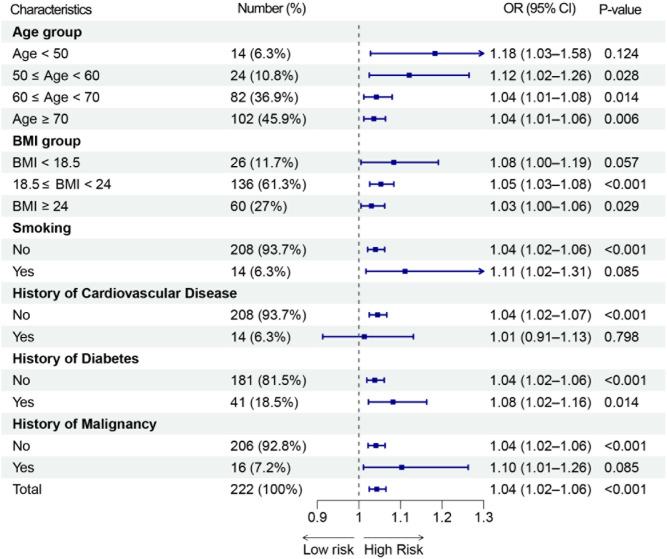
Forestplot of subgroup analysis for the association between 7dDDE and DVT.

It is worth noting that although the post-matching SMDs for history of malignancy and history of fracture remained slightly above 0.1, their prevalence in the cohort was relatively low (7.2% and 6.4%, respectively). These variables were included as covariates in the subsequent regression models to control for potential residual confounding. Additionally, in some subgroups such as patients aged < 50 years, those with a smoking history, or those with malignancy, the 95% confidence intervals for the odds ratios did not include 1. However, the *P* values did not reach statistical significance, which may be attributed to limited sample sizes in these subpopulations.

### Development and evaluation of the predictive model

**Fig. 3:**
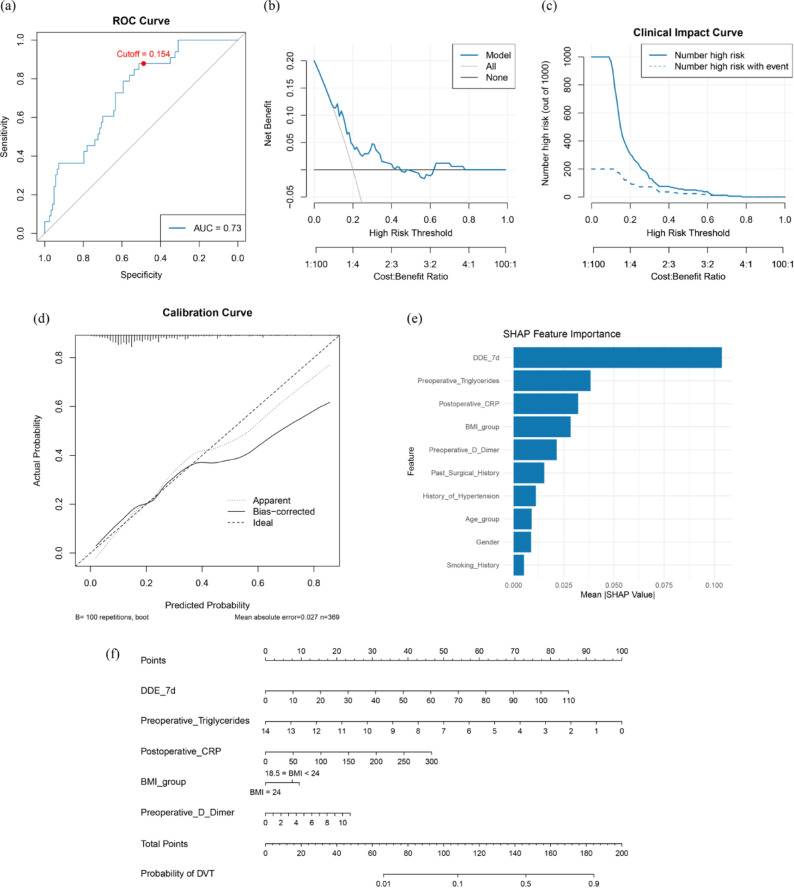
Model performance evaluation and interpretation in the testing set. (**a**) ROC curve of the logistic regression model incorporating 7dDDE. (**b**) Decision Curve Analysis showing the net clinical benefit of the model across a range of high-risk thresholds compared to “treat-all” and “treat-none” strategies. (**c**) Clinical Impact Curve displaying the number of individuals identified as high-risk and the number of true positives across different thresholds. (**d**) Calibration curve generated via 100 bootstrap resamples. (**e**) SHAP plot ranking feature importance. (**f**) Nomogram constructed based on the top five variables.

To evaluate the predictive ability for postoperative DVT, a multivariable logistic regression model was developed using the training set and validated in the testing set. The model achieved an area under the receiver operating characteristic curve of 0.73 in the testing set, indicating good discriminative performance. Based on the maximum Youden index, the optimal probability threshold was determined to be 0.154, yielding a sensitivity of 87.9% and a specificity of 51.2%. This cutoff point was marked with a red dot on the ROC curve (Fig. 3a), and incorporation of 7dDDE improved overall model performance in terms of AUC, sensitivity, balanced accuracy, and F1 score, with a statistically significant improvement in AUC (Supplementary Table 3).

DCA demonstrated that the model provided a greater net clinical benefit than the “treat-all” or “treat-none” strategies across a wide range of risk thresholds (Fig. 3b). The CIC further showed that the number of high-risk individuals predicted by the model closely matched the actual number of DVT cases at various threshold levels (Fig. 3c), supporting its potential clinical value for early postoperative risk stratification.

The model also exhibited good calibration. The calibration curve generated using 100 bootstrap resamples showed strong agreement between predicted and observed probabilities, with a mean absolute error of 0.027 (Fig. 3d). In the model interpretation analysis, SHAP value evaluation revealed that the five most influential variables were: 7dDDE, preoperative triglycerides, postoperative CRP, BMI group, and preoperative D-dimer (Fig. 3e). Based on these five predictors, a nomogram was constructed to facilitate individualized risk prediction and support clinical application (Fig. 3f).

### Longitudinal trend analysis of D-dimer by DVT status

To further explore the dynamic profile of D-dimer levels between the DVT and non-DVT groups, we fitted a linear mixed-effects model incorporating all five D-dimer timepoints (preoperative and postoperative days 1, 3, 5, and 7). After adjusting for age, BMI, gender, and log-transformed CRP, the interaction term between timepoint and DVT status was statistically significant (Supplementary Table 4), indicating that D-dimer levels followed distinct temporal trajectories in the two groups.

As shown in Supplementary Fig. 4, the DVT group exhibited persistently higher D-dimer levels at each postoperative timepoint compared to the non-DVT group. The model also identified age and CRP as independent predictors of elevated D-dimer. These findings suggest that the time-varying pattern of D-dimer, particularly in the early postoperative phase, differs substantially between patients with and without DVT, supporting the relevance of 7dDDE as a predictive metric.

## Discussion

DVT remains one of the most common and potentially fatal complications following gastrointestinal surgery, with the potential to cause pulmonary embolism and significantly increase perioperative morbidity and mortality. Although thromboprophylaxis strategies are widely implemented, timely identification of high-risk individuals remains challenging. D-dimer, despite its high sensitivity, has limited specificity^18^, and is commonly used as an auxiliary diagnostic indicator rather than a stand-alone predictor for DVT, particularly in postoperative settings^19,20^. While some non-surgical studies suggest that D-dimer may independently predict DVT and reduce the need for imaging^21^, its predictive value in the postoperative context remains suboptimal.

To address this gap, our team introduced a novel dynamic biomarker, termed 7dDDE, designed to quantify the cumulative coagulation burden in the early postoperative period. Unlike conventional models relying on single-time-point laboratory values or static baseline features, 7dDDE uses a time-weighted area under the curve approach to integrate both the magnitude and duration of D-dimer elevation, thereby reflecting the evolving coagulative status following surgery. This comprehensive metric potentially offers improved insight into the progression of postoperative hypercoagulability. In our study, 7dDDE was confirmed as an independent predictor of postoperative DVT, remaining statistically significant even after adjusting for confounders via PSM, and demonstrating consistent associations across most patient subgroups.

Furthermore, we constructed a logistic regression prediction model incorporating 7dDDE and validated its performance in an independent test cohort. The model exhibited good discriminative ability; during ROC analysis, we identified an optimal cutoff probability, above which patients can be classified as high-risk for postoperative DVT. For such individuals, intensified thromboprophylactic monitoring—such as dynamic D-dimer trend tracking, lower extremity Doppler ultrasound, or extended anticoagulation—may be considered. DCA and CIC supported its potential clinical utility. The model may serve as a clinical decision-support tool that can be embedded into perioperative care pathways, enabling individualized DVT risk prediction and helping clinicians optimize screening and intervention strategies in resource-limited settings. SHAP-based interpretability analysis identified 7dDDE as the most influential variable, followed by postoperative CRP, preoperative triglycerides, BMI group, and baseline D-dimer. These findings not only underscore the value of D-dimer kinetics but also highlight the complementary roles of inflammation, metabolic status, and individual physiological reserve in thrombotic risk prediction^22–24^.

In addition to the cumulative D-dimer exposure, our supplementary longitudinal analysis demonstrated that D-dimer trajectories were significantly different between patients with and without DVT. Even after adjusting for age, BMI, gender, and CRP, DVT patients exhibited consistently higher D-dimer levels across all postoperative timepoints. This reinforces the importance of capturing not only the absolute magnitude but also the temporal dynamics of coagulation biomarkers. Such findings lend further support to the validity of 7dDDE as a clinically meaningful and biologically plausible metric for thrombotic risk assessment in surgical populations.

From a pathophysiological perspective, the association between sustained D-dimer elevation and thrombosis risk is biologically plausible. D-dimer is a fibrin degradation product generated through a cascade involving thrombin formation, fibrin polymerization, and plasmin-mediated fibrinolysis. Elevated and prolonged D-dimer levels reflect ongoing thrombin activity and impaired fibrin clot resolution, both of which are hallmarks of a prothrombotic state^25^. Previous studies have demonstrated that postoperative D-dimer trajectories may offer more predictive value than single measurements. For instance, Palareti et al. reported that persistently elevated D-dimer after anticoagulation discontinuation was significantly associated with VTE recurrence^26^. Our study applies this concept to the immediate postoperative period by introducing 7dDDE, a quantifiable metric that reflects the dynamic coagulative burden. CRP, another key predictor in our model, serves as a surrogate marker for systemic inflammation and has been associated with endothelial dysfunction and thrombogenesis. Several epidemiologic studies have confirmed the link between elevated CRP and increased DVT risk^27,28^. Likewise, elevated triglycerides^29^ and higher BMI^30^ are well-established prothrombotic factors. The inclusion of these variables in our model, and their ranking in SHAP analysis, further supports the biological plausibility of the selected predictors. Importantly, our modeling approach also aimed to bridge statistical robustness and clinical usability. By translating the final model into a nomogram based on the top five predictors, we provide a simplified yet interpretable bedside tool for individualized DVT risk assessment, facilitating personalized perioperative management strategies.

Despite the innovative nature and clinical potential of this study, several limitations should be acknowledged. First, this was a single-center retrospective study conducted at a tertiary medical institution, which may limit the generalizability of our findings. Although we employed propensity score matching to control for key confounding variables and validated the model in an independent testing set, further external validation in multicenter or prospective cohorts is necessary to assess its robustness and applicability across broader populations. While multiple coagulation-related biomarkers were initially considered, they were not retained in the final model after feature selection. This does not imply a lack of clinical relevance but may reflect their relatively limited predictive contribution within our dataset. Additionally, although we used the fastshap method to enhance model interpretability and quantify the association between individual predictors and DVT risk, this approach is inherently correlational and does not infer causality. Moreover, although our model exhibited acceptable discrimination and calibration, the number of DVT events was relatively limited. Given the class imbalance and moderate sample size, potential overfitting cannot be entirely ruled out. While we explored SMOTE-based data augmentation, it did not significantly improve model performance. Therefore, the current findings should be regarded as preliminary, requiring validation in larger-scale datasets. Lastly, although comparing our model with established clinical tools such as the Caprini score would have provided meaningful clinical context, the retrospective nature of our dataset and the absence of key variables precluded this analysis. Future studies incorporating prospectively collected Caprini scores are warranted to better contextualize the added value of the proposed model.

## Conclusion

In this study, we introduced a novel cumulative biomarker, 7-day D-dimer exposure (7dDDE), to capture perioperative coagulation burden in gastrointestinal surgery patients. Our results demonstrated that 7dDDE is an independent predictor of postoperative DVT and the top contributor in a validated predictive model. Supplementary longitudinal analysis using a linear mixed-effects model further confirmed that D-dimer levels followed significantly different temporal patterns between DVT and non-DVT groups, even after adjusting for age, BMI, gender, and inflammation (CRP). This underscores the value of considering both the cumulative magnitude and dynamic trends of D-dimer when assessing thrombotic risk. Together, these findings support the integration of 7dDDE into perioperative risk assessment frameworks and highlight the need for further prospective studies to validate its predictive and mechanistic roles.

## Supplementary Information

Below is the link to the electronic supplementary material.

Supplementary Material 1

## Data Availability

Due to institutional and ethical restrictions, the datasets generated and analyzed during the current study are not publicly available. However, de-identified data and the R code used for data preprocessing, statistical analysis, and model development are available from the corresponding author upon reasonable request.
