# Supplementary material for: Integrating 7-day D-dimer exposure into deep vein thrombosis risk prediction after gastrointestinal surgery
Source: Sci Rep. 2025 Aug 13;15:29663. doi: 10.1038/s41598-025-14960-7 (PMC12350792; doi:10.1038/s41598-025-14960-7)
Supplement: Supplementary file 1 — Supplementary Material 1 [file 41598_2025_14960_MOESM1_ESM.docx]

**Supplementary Materials**

**
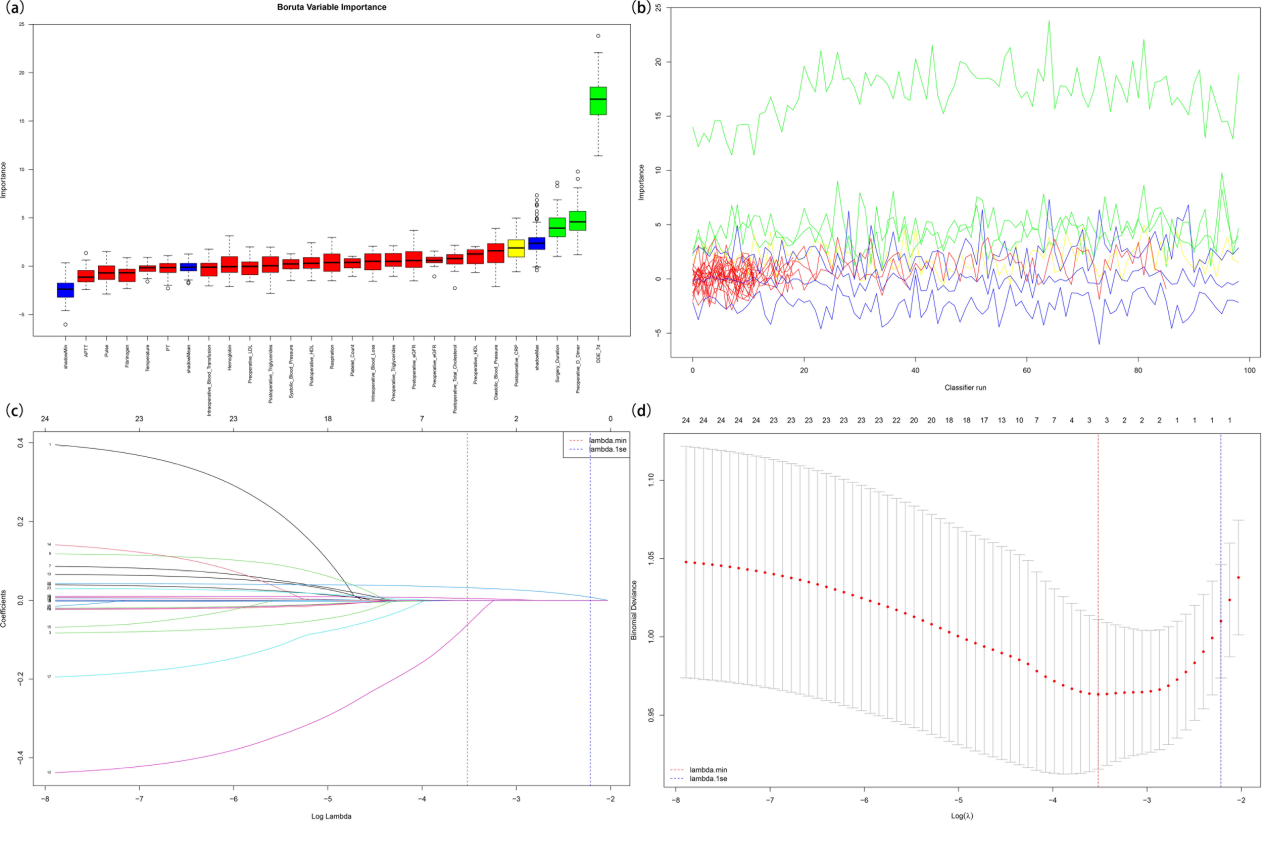
**

**Supplementary Figure 1.** Feature selection results using Boruta and LASSO algorithms.

Note: (a) Boruta feature importance plot, where green boxplots indicate confirmed important variables, red represent rejected variables, and blue indicate tentative ones. (b) Stability of variable importance scores across Boruta iterations, demonstrating the robustness of feature rankings. (c) LASSO coefficient profiles as a function of the regularization parameter (log λ), showing how coefficients shrink with increasing penalty.

(d) Ten-fold cross-validation for LASSO regression, identifying the optimal λ value (vertical dotted line) that minimizes the cross-validated error and determines the final set of selected features.


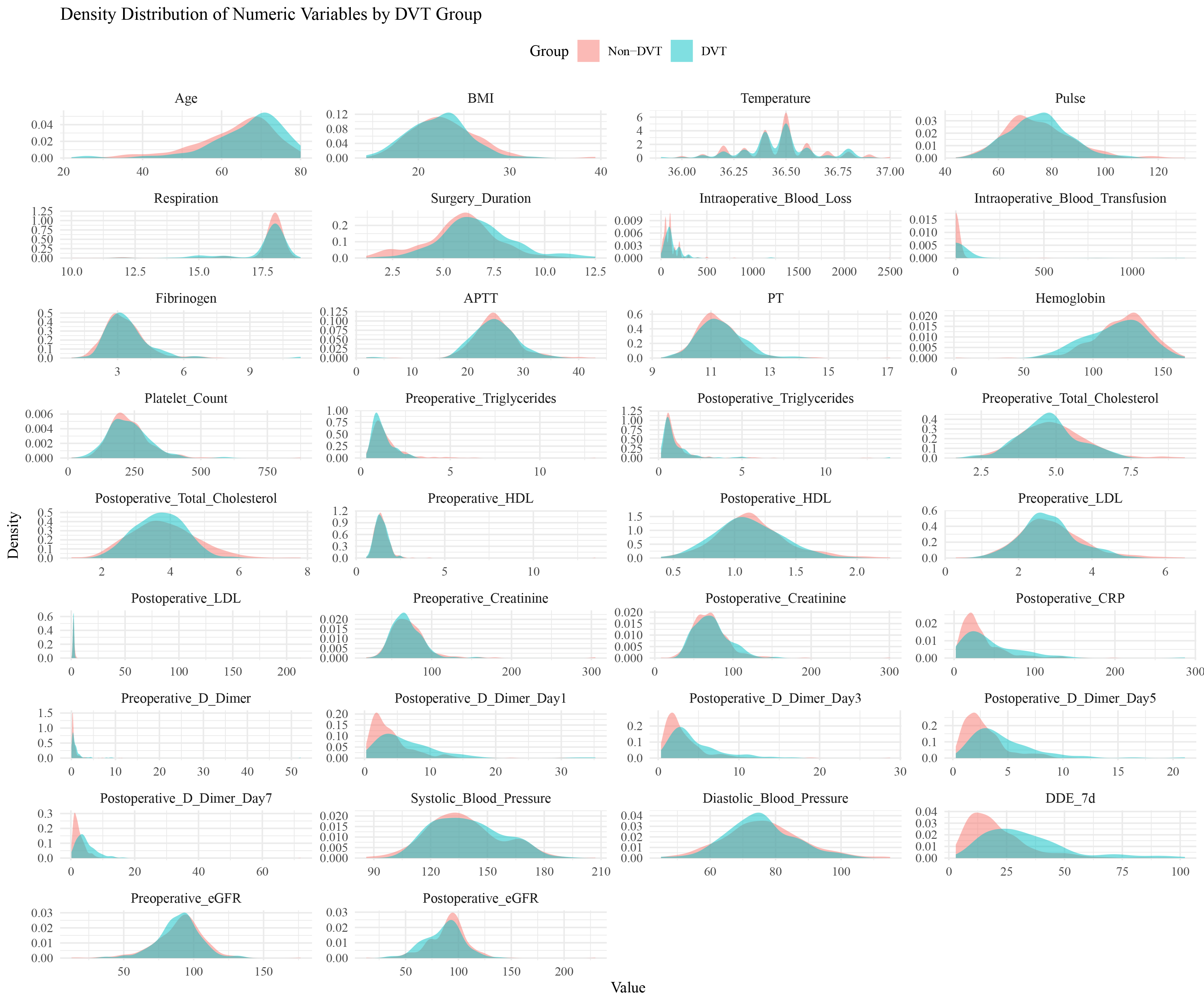


**Supplementary Figure 2.** Density distribution curves of all continuous variables in the DVT and non-DVT groups.





**Supplementary Figure 3.** Love plot showing covariate balance before and after propensity score matching.

Note: The figure presents standardized mean differences (SMDs) for key baseline covariates before (gray circles) and after (red triangles) PSM between DVT and non-DVT groups. Covariates include age group, BMI group, gender, and estimated surgical risk distance. The dashed vertical line at 0.1 denotes the commonly accepted threshold for adequate covariate balance. Post-matching, all covariates fall below this threshold, indicating successful adjustment and improved group comparability.





**Supplementary Figure 4.** Dynamic trajectories of D-dimer levels from baseline to postoperative day 7 between DVT and non-DVT groups.

Note: Adjusted for age, BMI, gender, and CRP. Error bars indicate standard error.

**Supplementary Table 1.** Feature engineering process for variable selection.

**Supplementary Table 2.** Subgroup analysis of 7dDDE and the DVT.

**Supplementary Table 3.** Comparison of model performance with and without 7dDDE in the testing set.

**Supplementary Table 4.** Type III analysis of variance (ANOVA) results from the linear mixed-effects model evaluating the temporal trend of D-dimer levels across five perioperative timepoints in DVT and non-DVT groups.
